# 320-row CT renal perfusion imaging in patients with aortic dissection: A preliminary study

**DOI:** 10.1371/journal.pone.0171235

**Published:** 2017-02-09

**Authors:** Dongting Liu, Jiayi Liu, Zhaoying Wen, Yu Li, Zhonghua Sun, Qin Xu, Zhanming Fan

**Affiliations:** 1 Department of Radiology, Beijing Anzhen Hospital, Capital Medical University, Beijing, China; 2 Department of Medical Radiation Sciences, Curtin University, Perth, Australia; 3 School of Public Health, Capital Medical University, Beijing, China; Universita degli Studi Magna Graecia di Catanzaro, ITALY

## Abstract

**Objective:**

To investigate the clinical value of renal perfusion imaging in patients with aortic dissection (AD) using 320-row computed tomography (CT), and to determine the relationship between renal CT perfusion imaging and various factors of aortic dissection.

**Methods:**

Forty-three patients with AD who underwent 320-row CT renal perfusion before operation were prospectively enrolled in this study. Diagnosis of AD was confirmed by transthoracic echocardiography. Blood flow (BF) of bilateral renal perfusion was measured and analyzed. CT perfusion imaging signs of AD in relation to the type of AD, number of entry tears and the false lumen thrombus were observed and compared.

**Results:**

The BF values of patients with type A AD were significantly lower than those of patients with type B AD (P = 0.004). No significant difference was found in the BF between different numbers of intimal tears (P = 0.288), but BF values were significantly higher in cases with a false lumen without thrombus and renal arteries arising from the true lumen than in those with thrombus (P = 0.036). The BF values measured between the true lumen, false lumen and overriding groups were different (P = 0.02), with the true lumen group having the highest. Also, the difference in BF values between true lumen and false lumen groups was statistically significant (P = 0.016), while no statistical significance was found in the other two groups (P > 0.05). The larger the size of intimal entry tears, the greater the BF values (P = 0.044).

**Conclusions:**

This study shows a direct correlation between renal CT perfusion changes and AD, with the size, number of intimal tears, different types of AD, different renal artery origins and false lumen thrombosis, significantly affecting the perfusion values.

## Introduction

Aortic dissection (AD) represents a common cardiovascular disease and it is responsible for the most commonly encountered pathologies in aortic emergency [1]. AD is characterized by separating the aortic wall layers into true and false lumens through an intimal entrance tear [2,3]. Renal dysfunction is a common complication associated with aortic dissection, with resultant high mortality rate of about 27.27% [4]. The antegrade propagation of the dissection from the proximal aorta to the level of renal arteries and the intervention during surgery may both increase the risk of renal malperfusion [5,6]; thus, it is important to assess renal function for pre-and post-operative evaluation and guidance for treatment. This can be achieved through perfusion imaging.

Computed tomography (CT) perfusion imaging can not only provide anatomical details, but also can describe vascular physiology [7,8]. A time-density curve can be calculated through dynamic CT imaging of tissue perfusion [9]. Presently, cerebral CT perfusion is well established, in particular, in the diagnostic assessment of acute stroke and tumors [10]; and the application of renal CT perfusion imaging is also widely used. There is increasing evidence to show that CT perfusion imaging is not only to measure changes in glomerular filtration [11] and evaluate angiogenesis [12], but also to monitor the response of tumors to treatment [13,14]. Several studies have demonstrated that multi-slice CT perfusion is a useful method for the evaluation of renal functions [8,15–17].

320-row CT is a recent technological development in CT imaging. Extended longitudinal coverage of 16 cm enables imaging of the entire kidney perfusion. To the best of our knowledge, no study has assessed renal CT perfusion using 320-row dynamic volume CT in patients with acute AD, so the aim of this study is to assess the clinical potential of using 320-row CT in evaluating renal function in patients with AD. We hypothesize that different variables of AD are closely correlated with changes in renal CT perfusion.

## Materials and methods

### Patient population

This prospective study was approved by the Institutional Review Board of Beijing Anzhen Hospital Ethics Committee. During the period from May 2014 to January 2016, 46 patients diagnosed with AD by transthoracic echocardiography in our department, none of whom had any history of impaired functions of the heart, liver, or kidneys, or a history of allergy to the iodine contrast medium, were invited to take part in this study. Further, patients who had accessory renal arteries were excluded from this study. Three cases were excluded from data analysis after CT imaging due to severe motion artifacts. The remaining 43 (35 males and 8 females) with a mean age of 50.47 ± 9.82 years (range 28–73 years) were included in the perfusion analysis. Written informed consent was obtained from all participants.

### CT contrast protocols

CT scanning was performed using a 320-row dynamic volume CT scanner (Aquilion One, Toshiba Medical Systems, Ottawara, Japan). The following contrast injection protocol was used for perfusion imaging: a bolus of iodinated contrast material 40 ml (Ultravist 370, 370 mg/mL; Bracco S.P.A., Italy) was administered intravenously at a flow rate of 5 mL/s, followed by a 20 mL saline chaser at the same flow rate. The total injection length was 8 s. Image post-processing was carried out on a Vitrea workstation with special CT body perfusion software.

### Procedure design and scanning techniques

In order to minimize the radiation dose, we established a dynamic CT protocol with the following parameters: tube voltage of 100 kV; tube current of 60 mA; 0.5 s gantry rotation time; 1 mm pixel spacing; 512 X 512 pixel (spatial resolution); and 0.5 mm reconstructed slice thickness.

Instructions were given to patients prior to CT scans to ensure natural breathing. The first and second steps were similar to a previous study [18], then they were followed by a single-section tracking series (section thickness, 5 mm; tube current, 60 mA; tube voltage, 100 kV) at the renal hilum level. In the third step, volumetric cine scans were automatically triggered at a preset threshold of 90 HU in the lumen of the abdominal aorta above the renal artery. In total, 17 CT volumes of the kidneys were acquired without table movement. It took 0.5 s to complete each of these 17 scans (0.5 s per gantry rotation of 360 degree for one volume data acquisition on 320-slice Toshiba scanner, with different temporal sampling intervals being 1.5 to 4.5 s), thus the whole process took 58 s in total. The data were processed with adaptive iterative dose reduction and were reconstructed automatically with a section thickness of 5 mm and 5 mm spacing.

The volumetric CT dose index (CTDI_vol_) and dose-length product (DLP) of CT examination was automatically recorded and displayed on CT console. The effective dose (ED) was estimated using an organ conversion coefficient k-factor of 0.015 mSv/mGy.cm [19].

### Perfusion image post-processing

**Fig 1:**
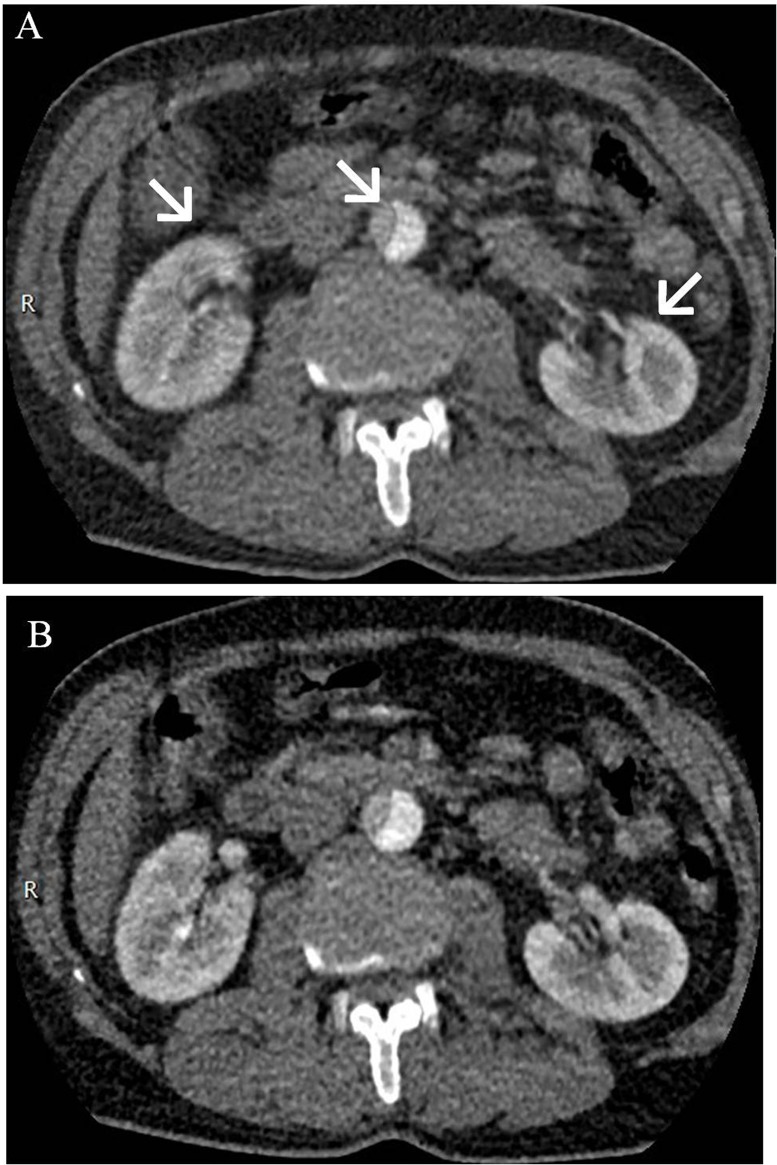
Transverse contrast-enhanced CT scan showed the effect of motion correction. (A) Motion artifacts were seen as blurring of the kidney and aorta contours (white arrowheads). (B) Most motion artifacts were not seen after correction.

Post-processing and measurement of CT perfusion images were conducted by two experienced radiologists with 12 and 21 years of diagnostic experience in CT imaging, who reached consensus on the image analysis. We used the same approach as reported in a previous study to deal with motion and breathing artifacts [18]. Fig 1 is an example showing image registration processing.

**Fig 2:**
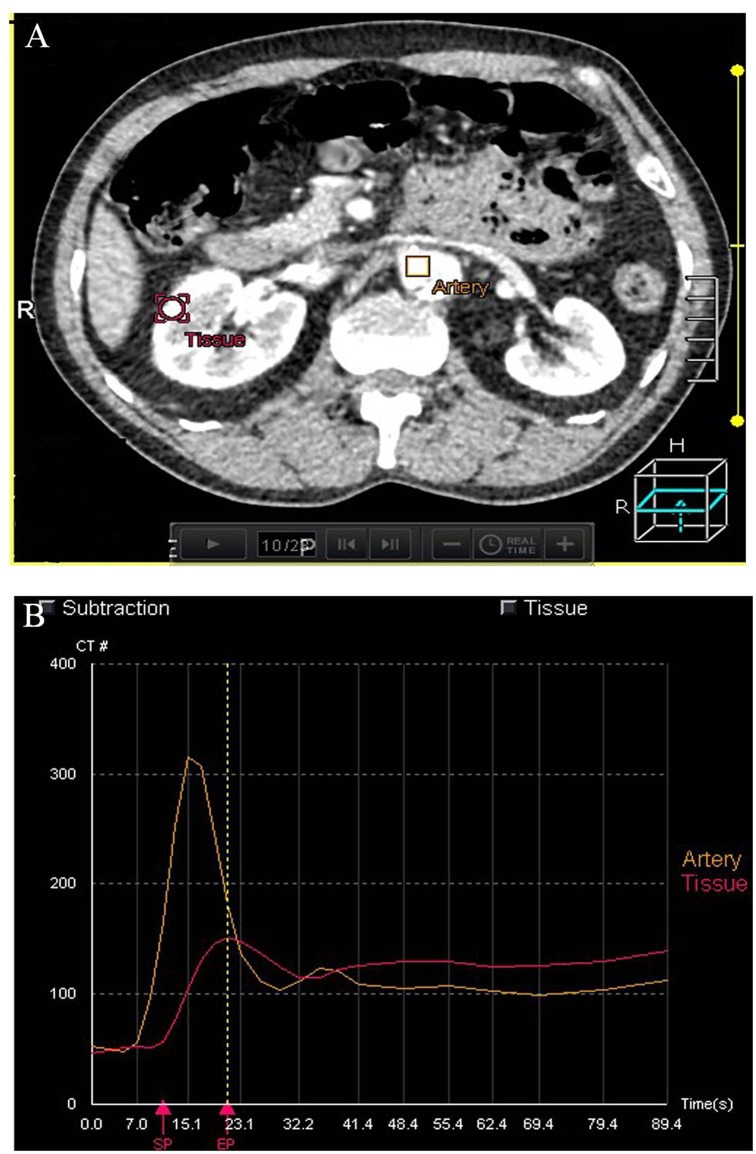
Time—density curves (TDCs) of renal cortex from the single input maximum slope. (A) The ROIs were placed in the abdominal aorta and renal cortex. (B) TDC indicates the enhancement characteristics of the aorta and renal cortex during the first pass. Blood flow (BF) can be determined from the maximum gradient of renal cortical TDC by the peak enhancement of the aorta.

The new registered datasets were post-processed using Body Perfusion software (Vitrea fx ves 6.0, Licensed software, Toshiba Medical Systems) with details as follows: The region of interest (ROI) was placed in the abdominal aorta, renal cortex separately at the level of the renal hilum to obtain a time-density curve (TDC). A previously reported model was used [20] to acquire a blood flow (BF) map (BF mL/ min /100 mL) based on TDC (Fig 2).

**Fig 3:**
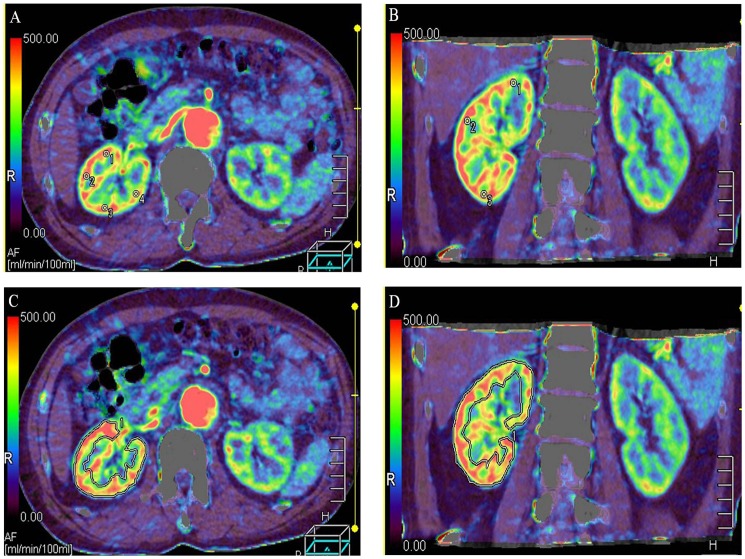
Examples of regions of interest (ROIs) measurements. (A) ROIs of renal cortex at axial view. (B) ROIs of renal cortex at coronal view. (C) ROIs of whole kidney were defined manually on the axial plane. (D) ROIs of whole kidney on the coronal plane.

The perfusion maps (Fig 3) were generated by the Body Perfusion software. The ROIs of the renal cortex were selected from four points on the axial section of kidney in an X shape; three points of upper pole, hilum and lower pole of kidney at coronal section separately (Fig 3A and 3B) and whole kidney (Fig 3C and 3D) were defined manually on the axial and coronal planes. The BF values were recorded separately and the BF values of the different types of aortic dissection were compared.

**Fig 4:**
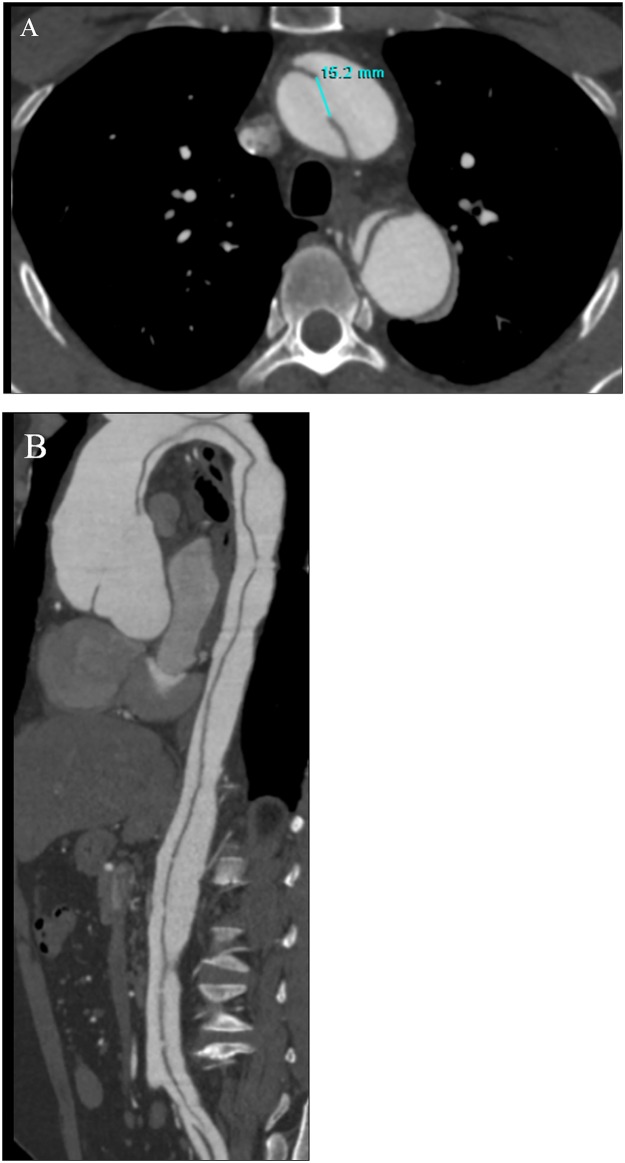
Example of size measurement and numbers of the entry tears. (A) Primary entry tear located in the aortic arch. (B) MPR image displaying five small re-entry tears in descending and abdominal aorta, with the primary entry tear locate in the ascending aorta.

For all patients, dynamic CT examinations were executed on thoracic and abdominal great vessels, and multiplanar reconstruction (MPR), maximum-intensity projection (MIP) and volume rendering (VR) images were generated to observe these various CT imaging signs associated with AD, which included different AD types, origin of renal arteries, the false lumen thrombosis, and the size, number and position of entry tears. A comparison was then performed between the perfusion imaging and these dissection features. We measured the size, number and position of entry tears on MPR reconstruction images (Fig 4A and 4B). Based on different MPR images, we choose the maximum diameter as the size of the entry tears.

### Statistical analysis

Statistical analysis was performed using SPSS (SPSS, V 20.0A IBM, Armonk, NY, USA). Continuous variables of perfusion parameters were presented as mean and standard deviation (mean ± SD). Independent-Samples T test was used to analyze the relationship of renal perfusion to the number of intimal entries and different AD types, and to assess the relationship of false lumen thrombosis and BF. Analysis of variance (ANOVA) was used to evaluate the differences of the perfusion values among the three groups of renal arteries: originating from the true lumen, from the false lumen, or overriding. A simple regression analysis was performed to examine the relationship between the BF and the size of intimal entries. Multiple linear regression was used to assess the relationships between various kinds of variable and the BF. The axial BF and coronal BF values of the same patient were assessed using Bland-Altman plots: a p value less than 0.05 was considered to indicate a significant difference.

## Results

The perfusion imaging examinations were successfully performed in all 43 patients with no technical problems or adverse reactions to the contrast medium. The estimated mean ED was 11.09 ± 0.29 mSv (range, 10.69–11.72 mSv). Type A AD was found in seven patients with the intimal tear located in the ascending aorta, while type B AD was found in 36 patients with the tear located distal to the left subclavian artery orifice or descending aorta. Twenty-two patients had one entry, and the remaining 21 had two or more (3 to 6) entries. Of the 86 renal arteries analyzed, 58 originated from the true lumen, 20 from the false lumen; 8 were overriding (Table 1). Patients with accessory renal arteries were not included in the analysis due to potential bias of renal perfusion analysis. (S1 File)

**Table 1:** Characteristics of study population.

Characteristic		N (%) *	Mean ± SD ^#^
Gender	Male	35 (81.4)	—
	Female	8 (18.6)	—
Age		—	50.47±9.82
AD type	A	7 (16.3)	—
	B	36 (83.7)	—
Number of entries	1	22 (51.2)	—
	2	13 (30.2)	—
	3	4 (9.3)	—
	4	1 (2.3)	—
	5	2 (4.7)	—
	6	1 (2.3)	—
Size of entries (mm)	1	—	12.57±8.13
	2	—	6.57±4.57
	3	—	4.37±1.70
	4	—	5.60±2.66
	5	—	6.35±1.60
	6	—	5.00±0.00
Thrombus	No	37 (86.0)	—
	Yes	6 (14.0)	—
Origin	True lumen	58(67.4)	—
	False Lumen	20 (23.2)	—
	Overriding	8 (9.3)	—

* N indicates the number of patients, and data in parentheses are percentages.

^#^ Represents mean ± standard deviation.

Number of entries and Size of entries, 1, one intimal tear; 2, one entry tear and one secondary tear; 3, one entry tear and two secondary tears; 4, one entry tear and three secondary tears; 5, one entry tear and four secondary tears; 6, one entry tear and five secondary tears. Abbreviation: AD, aorta dissection.

### CT color maps

**Fig 5:**
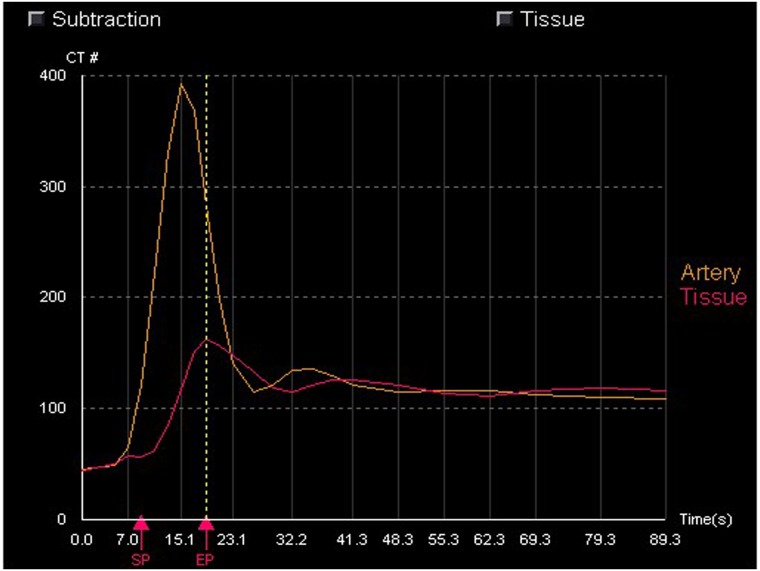
Time—density curves (TDCs) from the single input maximum slope. TDC indicates the enhancement characteristics of the aorta and renal cortex during the first pass. BF can be determined from the maximum gradient of renal cortex.

**Fig 6:**
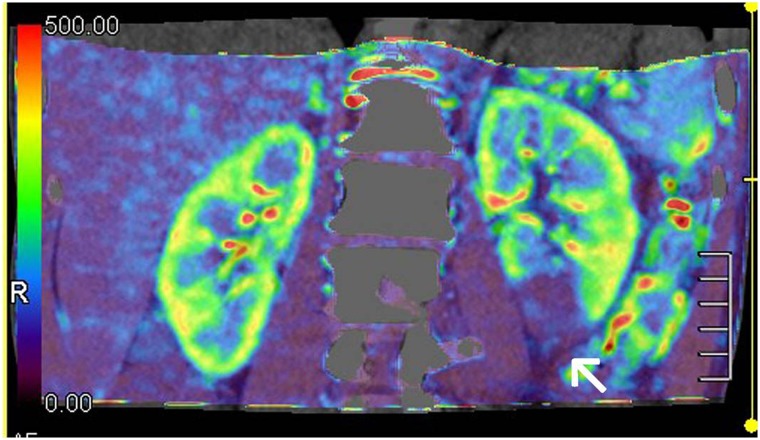
Blood flow (BF) color maps of bilateral kidneys. White arrow indicate the partially decreasing BF.

The TDC curves of the renal tissue in AD demonstrate slowly upgrading slopes, lower peak values and delayed time to peak (TTP) (Fig 5). The color maps of the kidney reveal that the renal cortex has become thin and BF has decreased. In three cases the BF color maps are inhomogeneous, indicating the possibility of a partially decreasing BF. One patient in this group shows infarction at the inferior pole of the left kidney (Fig 6).

### Mean perfusion parameters of bilateral kidneys in AD

The average BF values of two types of AD, the different number of intimal tears and the false lumen with or without thrombosis, are summarized in Table 2. The BF values of patients with type A AD were significantly lower than those of patients with type B AD (P = 0.004). No significant difference was found between BF values and the number of intimal tears (P = 0.288). The BF value of AD with no thrombus in the false lumen was significantly higher than that of the false lumen with thrombus (P = 0.036). Different renal artery origins caused different BF values. The BF values among the true lumen, false lumen and overriding groups were different (P = 0.02): with the true lumen group having highest, and the overriding group lowest. The BF values between the true lumen and false lumen groups were significantly different (P = 0.016), but there was no statistical significance between the false lumen and overriding groups, and the true lumen and overriding groups (P > 0.05), with findings summarized in Tables 3 and 4.

**Table 2:** Independent-Samples T test.

Variables		BF		*t* value	*P* value
		Mean	SD		
AD type	A	218.82	47.01	-3.040	0.004
	B	270.74	98.19		
Number of tears	1	251.76	95.65	-1.069	0.288
	≥2	273.32	91.25		
Thrombus	No	270.79	96.51	2.13	0.036
	Yes	209.88	49.07		

AD-aortic dissection, BF-blood flow, BF: mL/ min /100 ml, SD-standard deviation.

**Table 3:** One-way ANOVA.

Variables	Mean	Std. Deviation	*F* value	*P* value
True lumen	281.464	83.692	4.01	0.02
False lumen	223.905	90.281		
Overriding	219.237	103.652		

α = 0.05

**Table 4:** Multiple comparisons (LSD-t test).

	Mean difference (95%CI)	Std. Error	*P* value
True lumen vs False lumen	57.558 (10.904 to 104.213)	23.46	0.016
False lumen vs Overriding	4.667 (-70.598 to 79.933)	37.84	0.902
Overriding vs True lumen	-62.22629 (-130.082 to 5.630)	34.12	0.072

α = 0.05/3

**Fig 7:**
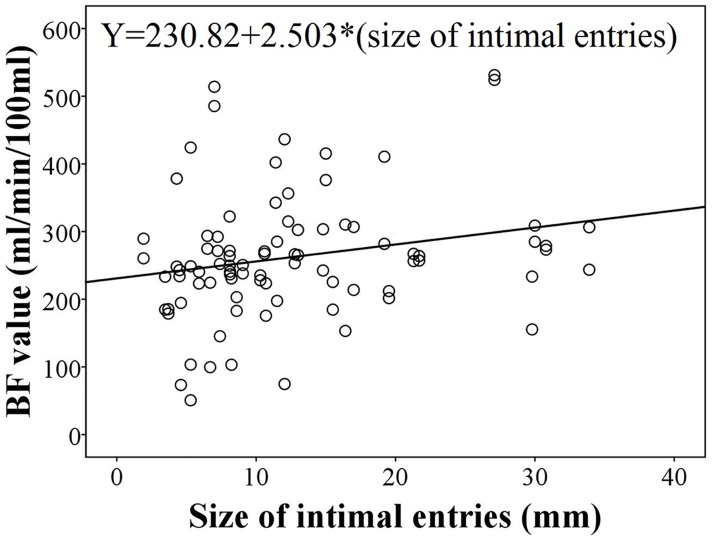
Result of simple regression analysis. The result indicates the positive correlative relationship between the perfusion values and the size of intimal entries.

The size of the intimal entry tears has a direct relationship with BF. The regression coefficient is 2.503 (95% CI: 0.063–4.942). A coefficient greater than 0 indicates the size of intimal tears has a positive correlative relationship with BF. With the increasing size of intimal entry tears, the BF value of the kidney increased significantly (P = 0.044, Table 5), result of regression equation was shown in Fig 7.

**Table 5:** Linear regression results for the effect of the size of the intimal entry on BF.

	Sum of Squares	df	Mean Square	*F* value	*P* value
Regression	35160.257	1	35160.257	4.162	0.044
Residual	709633.723	84	8448.021		
Total	744793.981	85			

BF-blood flow

**Fig 8:**
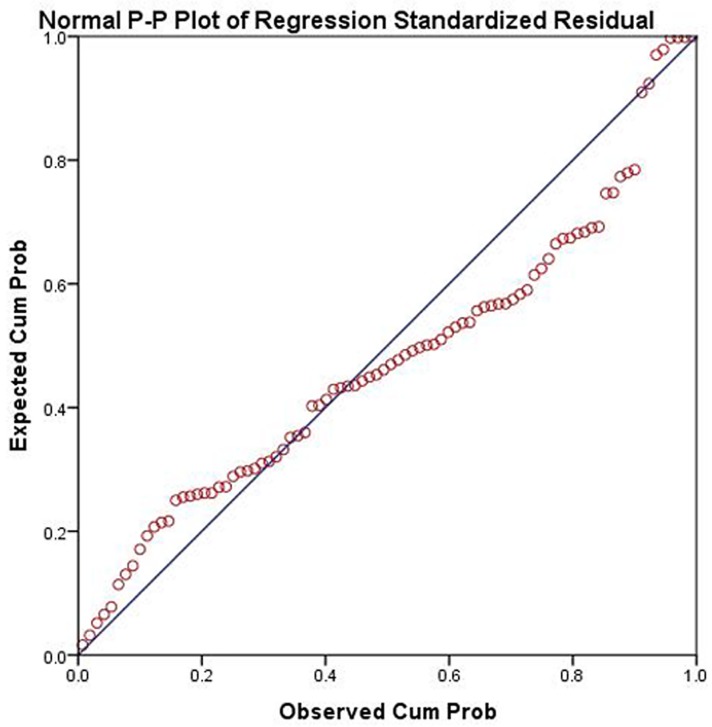
Residual diagram of model fitting of multi factor regression analysis of BF value. The result displays all the points of distribution on both sides of central line, the residual plots obey normal distribution.

The results of multiple linear regression analysis of the 86 renal arteries from 43 patients including age, gender, type and other factors showed that the higher the numbers of intimal entry tears, false lumen without thrombosis, and renal artery originating from the true lumen, the higher the BF values. The BF value of renal perfusion was significantly related to the number of intimal tears, the false lumen with or without thrombosis, and origin of the renal artery (P < 0.0001). Fig 8 presents model fit residual plots following normal distribution, showing that the model fits well.

**Fig 9:**
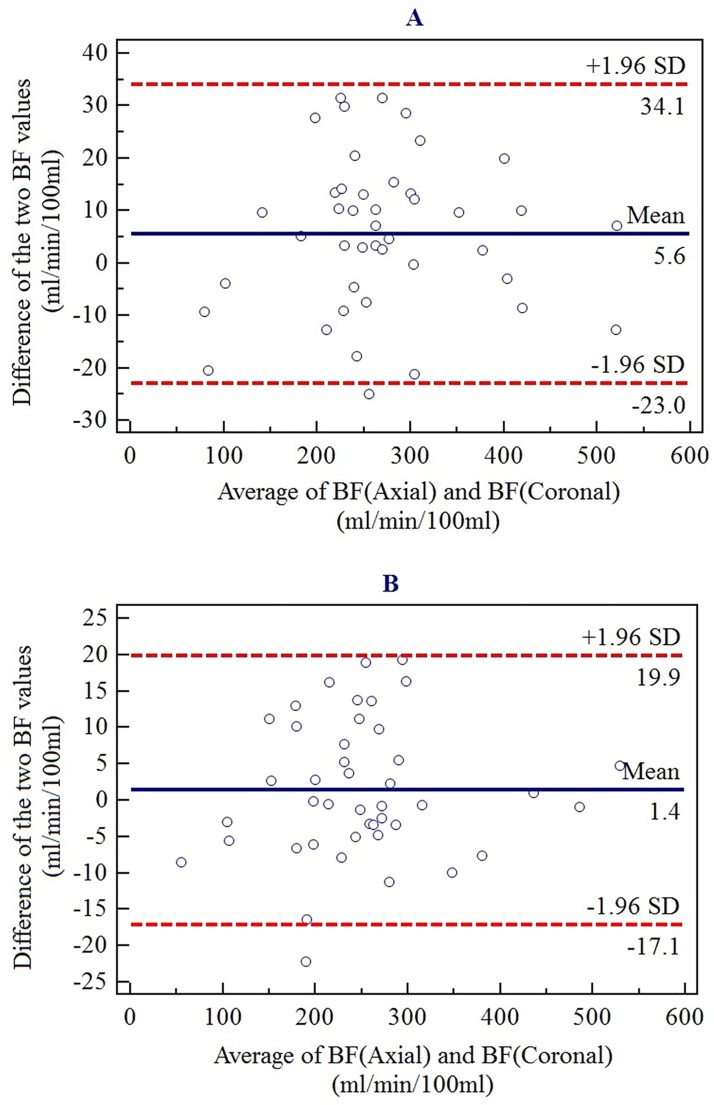
Bland-Altman analysis on differences between the average blood flow (BF) value of the axial plane and that of the coronal plane. (A) BF value differences between the two planes for right kidney.(B) BF value differences between the two planes for left kidney. Mean difference (blue line), mean difference ± 1.96 SD (red line).

Bland-Altman analysis for differences in BF value from two different observation positions (Fig 9A and 9B) reveals that the BF value of the axial section is similar to that of the coronal section in each patient. The analysis was performed on the left and right sides of 86 renal arteries in 43 patients, with good consistency.

## Discussion

In this study we investigate the clinical application of renal perfusion in patients with aortic dissection. Our findings show that blood flow values to the kidneys are directly related to the type of aortic dissection, renal artery origins, the number and size of the intimal tears, and to false lumen thrombosis.

CT perfusion, a non-invasive method for hemodynamic and functional evaluation of tissues and organs, can provide organ anatomy and quantitative perfusion parameters in one scan. The theoretical base of CT perfusion is radioactive tracing (from nuclear medicine) and central volume discipline; the application base is the rapid volume scanning technique of a multi-detector spiral CT [21]. CT perfusion is performed by scanning the same slice or volume at different times after an intravenous injection of a contrast medium, to get the TDC curve of each pixel in the selected slice or volume; perfusion parameters are then calculated according to different algorithms. Renal CT perfusion study without 320-row CT has several limitations in clinical use: first, the longitudinal scan range is limited, with the ROI full definition set at the level of the renal hilum and is unable to reflect the whole kidney perfusion (the upper and lower poles of the kidney). Second, it requires strict breath holding; but for elderly, severely ill, or pediatric patients, holding breath for more than 60 seconds presents challenges and is impossible in some cases. This presents a problem as the target region of the kidney may exceed the image plane in such cases. It is also difficult to obtain satisfactory images and data because of motion artifacts and image mis-registration [18]. The 320-row CT can avoid motion artifacts because of its high temporal resolution and use of image registration post-processing software [22].

Hemodynamic changes in tissues or local lesions have been evaluated by these perfusion parameters and color maps [23–25]. Daghini et al. [26] studied renal hemodynamic and function changes in stenotic renal arteries in animal models by analyzing renal perfusion compared with that of full-definition electron beam computed tomography(EBCT). They concluded that the multi-slice CT renal perfusion can accurately assess quantitative renal hemodynamic and functional changes. Blood flow was defined as the flow rate through the vasculature in a given tissue region, an important parameter in CT perfusion study equal to blood perfusion volume [27,28]. These studies show the feasibility of renal CT perfusion in renal artery disease; however, the current study utilizes the technique in aortic dissection, which has not been investigated before.

Assessment of organ or tumor perfusion has been of interest for years. In the brain, CT perfusion imaging plays an important role from the clinical perspective, detecting ischemic tissue in patients with acute stroke [29]. Also, it demonstrates hemodynamic changes in brain tissue after acute supratentorial spontaneous intracerebral hemorrhage [30]. Perfusion CT can also be applied to the diagnosis and monitoring of renal tumor and liver carcinoma [31,32]. In our study, 320-row dynamic volume CT successfully assessed bilateral renal imaging. In AD patients, The TTPs of aorta and kidney are all delayed, and TDC curves of the false lumen have lower peak values and lower upgrade slopes. Our results demonstrate a decrease in the perfusion parameters of the AD kidneys, which represents an abnormal low perfusion in the cortex and medulla in patients to different degrees. In our study, average BF values in the true lumen, the false lumen and the overriding groups were 281.46, 223.90 and 219.24mL/100mL/min respectively, which are lower than the values of normal kidneys reported in previous studies using the same CT system [18,33]. Zhong et al. [33] reported that the BF values of the left and right renal cortex were 323.8 and 322.9 mL/100mL/min in control group. Chen et al. [18] reported that the BF value of the normal renal cortex was 305.4 mL/100mL/min. The BF values of the coronal section are similar to those of the axial section in our study.

A study by Kandel et al. [34] reported that a relatively slow injection rate of 11.5 seconds in a maximum slope perfusion CT protocol could lead to an 8% underestimation of true perfusion. Yuan et al. [35] showed that using the maximum slope method as a reference standard was likely to cause a slight underestimation of the true perfusion value because of the long injection duration of the protocol. In our study, the use of 40 mL contrast agent within eight seconds of the injection guaranteed the accuracy of the perfusion value. This is clinically important, as reducing the contrast medium contributes to the reduction of contrast-induced acute kidney injury, the main concern associated with contrast-enhanced CT imaging.

Studies have found that patients with AD generally present more than two tears [36]. These secondary tears are typically found in the vicinity of visceral branches, and especially near the opening of the renal artery. It remains an important question whether secondary tearing has a significant impact on the dissection hemodynamic; thus in our study, including gender, age, and the size, number and position of intimal entries and a variety of other factors were considered in the multiple linear regression analysis. It was found in our study that the number of intimal tears, the false lumen thrombosis and different renal artery origin have important influence on the BF value. We believe these findings could assist in the assessment of renal function for pre- and post-operative evaluation and treatment guiding.

Acute AD is one of the most lethal surgical emergencies of the aorta, resulting from a tear in the aortic wall intima and extending into the aortic wall media to create a false lumen and a dissection flap [37]. Determination of renal cortex blood perfusion is a direct index by which to judge renal blood flow. In many pathological conditions such as acute and chronic renal failure, nephritis, urinary system obstruction, renal transplant rejection, renal artery stenosis and other diseases can affect the renal cortex and medulla perfusion [9,38,39]. Many studies have considered perfusion in this field, but renal blood perfusion changes in patients with AD have rarely been reported. Nearly 30% of acute AD associated with branch artery ischemia and renal artery involvement lead to renal blood perfusion changes. It has been found that the AD postoperative mortality rate is significantly associated with the duration of preoperative ischemic symptoms. Organ blood flow changes should be observed closely for AD patients. If this is delayed for a long time for an emergency operation induced by organ ischemia, multi-organ failure cannot be reversed even if blood flow is recovered. A study has shown that half of the aneurysmal patients with type B dissection who died after operations experienced partial irreversible damage before interventional therapies [40]. As CT perfusion imaging can reveal the hemodynamic changes of the kidney in AD patients, the findings of this study will assist clinicians to predict renal blood flow changes and so minimize mortality. Renal perfusion imaging can be used to guide clinicians choose surgery time, for example, patients with lower BF should be operated as soon as possible while higher BF would allow more time for thorough pre-operative planning. Both of surgical and interventional procedures may further affect the renal function. For patients with type A AD, if renal artery originates from the false lumen with lower BF, surgical procedure is recommended. Surgeons need to pay more attention to the involved renal artery during operation because acute kidney injury is one of the common complications that may occur after operation. CT renal perfusion imaging is a useful tool to assess renal functional status pre-operation, reveal the situation of renal ischemia post-operation, and early positive therapy can prevent the development of renal failure. We believe that CT-derived BF measurement could have a prognostic value for AD patients, although further studies with inclusion of large cohort of patients are needed.

Some limitations in this study should be addressed. First, the sample size is small, and is also based on a single-center experience. Further studies based on the analysis of more cases are required. Second, we did not study renal function changes, although no impairment was found in the recruited patients. Longitudinal studies with inclusion of follow-up data would correlate CT perfusion imaging with clinical outcomes, in particular with changes in renal function. Third, since patients with accessory renal artery were not included, their results may not apply to those patient presenting an accessory renal artery. Finally, although CT perfusion imaging in this study afforded statistical power to detect significant changes in renal perfusion in patients with different types of AD, correlation of CT perfusion imaging with CT-derived computational fluid dynamics (CFD) could represent further research investigations. CFD is increasingly used in cardiovascular disease due to its ability of quantifying hemodynamic changes to the aortic dissection and coronary artery disease with promising results reported [41,42]. In particular, CFD simulations of aortic dissection pre- and post-endovascular stent grafting have been shown to play an important role in the prediction of disease progression and patient outcome [42].

## Conclusion

This study shows that 320-row dynamic volume CT brings an unprecedented improvement in renal perfusion and provides important information about whole kidney blood perfusion in aortic dissection patients. It is concluded that CT perfusion imaging is an effective and non-invasive technique to evaluate renal perfusion in aortic dissection patients.

## Supporting information

S1 File (XLSX)

## Abbreviations

AD: aortic dissection
ANOVA: analysis of variance
BF: blood flow
CM: contrast media
CT: computed tomography
CTDI: computed tomography dose index
DLP: dose-length product
ED: effective dose
FOV: field of view
HU: Hounsfield unit
MIP: maximum intensity projection
MPR: multi planar reconstruction
ROI: region of interest
SD: standard deviation
TDC: time-density curve
TTP: time to peak
VR: volume rendering
